# Whole-Genome Sequence of *Pseudomonas putida* Strain UASWS0946, a Highly Ammonia-Tolerant Nitrifying Bacterium Isolated from Sewage Sludge Aerobic Granules

**DOI:** 10.1128/genomeA.01153-15

**Published:** 2015-10-08

**Authors:** Julien Crovadore, Gautier Calmin, Bastien Cochard, Romain Chablais, Damien Grizard, Jean-Yves Berthon, François Lefort

**Affiliations:** a Plants and Pathogens Group, Research Institute Land Nature and Environment, hepia, HES-SO University of Applied Sciences and Arts Western Switzerland, Geneva, Switzerland; b Faculty of Engineering and Architecture, HES-SO University of Applied Sciences and Arts Western Switzerland, Delémont, Switzerland; c Biovitis SA, St-Etienne-de-Chomeil, France; d Greentech SA, Saint-Beauzire, France

## Abstract

We report here the genome of *Pseudomonas putida* strain UASWS0946, a highly ammonia-tolerant nitrifying strain isolated from sewage sludge aerobic granules, which displays adequate genetic equipment for soil depollution, sludge treatment, and biological fertilization in agriculture.

## GENOME ANNOUNCEMENT

*Pseudomonas putida* is a Gram-negative saprotrophic and innocuous bacterium and a resident of various environments such as soil, water, and plant rhizospheres. It is known for displaying important metabolic functions in the biodegradation of aliphatic and aromatic compounds and soil bioremediation (1, 2). Some strains have been reported for their efficiency as biocontrol agents against plant pathogens and nematodes, as well as for their plant growth–promoting effects (3–7).

The strain UASWS0946 was isolated from a sample of sewage sludge aerobic granules in an experiment to select highly ammonia-tolerant nitrifying bacteria, and its identity was confirmed by 16S sequencing. Genomic DNA was extracted from pure axenic cultures grown to the stationary phase following an adapted protocol (8). Libraries were generated using the Nextera XT kit (Illumina, USA). Whole-genome shotgun sequencing was carried out within one Illumina MiSeq run at 2 × 250-bp read length, using the MiSeq reagent kit version 2 (Illumina) and yielded 191 contigs, providing a 42× genome coverage for a total genome length of 6,001,296 bp, with a GC content of 63.9% and a scaffold *N*_50_ value of 103,815 bp. Raw reads were trimmed with FastQC (9) and assembled with the SPAdes genome assembler version 3.1.1 (10). Resulting contigs of the genome assembly were arranged with BioEdit (11) and analyzed with QUAST (12). *In silico* screening with PlasmidFinder (13) did not identify any circular or integrated plasmid genome. Automated gene annotation was carried out by the NCBI Prokaryotic Genome Automatic Annotation Pipeline (PGAAP) (14) and reviewed with RAST version 2.0 (15). It allowed for the identification of 5,438 genes distributed in 5,179 coding sequences (CDSs), 189 pseudogenes, 4 rRNA genes (5S, 16S, 23S), 62 tRNAs, 4 ncRNAs, and 18 frameshifted genes. RAST version 2.0 analysis identified 5,628 CDSs, 46% of which could be allocated a function in a given subsystem. No transposon was detected, but 58 phage-related sequences were found to be integrated. Genes of toxins and superantigens, as well as genes of virulence and disease are absent, which will allow this bacterium to be considered as a biological fertilizer in agriculture and soil depollution. Additionally, it is equipped with 15 genes for bacteriocin and antimicrobial synthesis, as well as 112 genes involved in antibiotics and toxic compounds resistance. The bacterium is fully equipped for ammonia assimilation. Five genes are involved in plant auxin synthesis, which could be the basis for the growth-promoting properties of this bacterium. Similar to many strains of this species (16–18), UASWS0946 harbors many metabolic pathways for the degradation of aromatic compounds, with 102 genes involved in common pathways such as the central metacleavage pathway of aromatic compound degradation, as well as for the degradation of quinate, *n*-phenylalkanoic acid, benzoate, *p*-hydroxybenzoate, beta-ketoadipate, 4-hydroxyphenylacetic acid, homogentisate, gentisate, chloroaromatic, and N-heterocyclic aromatic compounds. It also showed a very high flocculating activity in a pilot wastewater reactor, and preliminary tests on plants confirmed plant growth–promoting activity. An extended comparison of this genome to similar genomes would allow a better understanding of its metabolic properties, which would make this strain a potential biological fertilizing agent and a tool for wastewater management.

### Nucleotide sequence accession numbers.

This whole-genome shotgun project was deposited at DDBJ/EMBL/GenBank under the accession number JXOG00000000. The version described in this paper is the first version, JXOG00000000.1. The 191 contigs have been deposited under the accession numbers JXOG01000001 to JXOG01000191.
